# EZH2: Its regulation and roles in immune disturbance of SLE

**DOI:** 10.3389/fphar.2022.1002741

**Published:** 2022-10-13

**Authors:** Yiying Yang, Ke Liu, Meidong Liu, Huali Zhang, Muyao Guo

**Affiliations:** ^1^ Department of Rheumatology, Xiangya Hospital, Central South University, Changsha, China; ^2^ Department of Pathophysiology, School of Basic Medicine Science, Central South University, Changsha, China; ^3^ Sepsis Translational Medicine Key Lab of Hunan Province, China; ^4^ Provincial Clinical Research Center for Rheumatic and Immunologic Diseases, Xiangya Hospital, Changsha, China; ^5^ National Clinical Research Center for Geriatric Disorders, Xiangya Hospital, Changsha, China

**Keywords:** EZH2, systemic lupus erythematosus, T cells, B cells, immune homeostasis

## Abstract

The pathogenesis of systemic lupus erythematosus (SLE) is related to immune homeostasis imbalance. Epigenetic mechanisms have played a significant role in breaking immune tolerance. Enhancer of zeste homolog 2 (EZH2), the specific methylation transferase of lysine at position 27 of histone 3, is currently found to participate in the pathogenesis of SLE through affecting multiple components of the immune system. This review mainly expounds the mechanisms underlying EZH2-mediated disruption of immune homeostasis in SLE patients, hoping to provide new ideas in the pathogenesis of SLE and new targets for future treatment.

## 1 Introduction

Systemic lupus erythematosus (SLE), is a highly heterogeneous autoimmune disease, characterized by damage of multiple systems and extensive presence of autoantibodies (Rapoport and Bloch, 2012). The occurrence of SLE is related to the excessive immune response to self-antigens and the lack of immune suppression, which in turn leads to a breakdown of immune tolerance. Nonetheless, the direct etiology of SLE is still not clear. Many factors are involved in the pathogenesis, including genetics, epigenetics, and environmental factors (Weeding and Sawalha, 2018; Xu et al., 2019).

Nowadays, epigenetic mechanisms have gradually been recognized as crucial factors in SLE pathogenesis, and epigenetics has become a focus of research (Teruel and Sawalha, 2017; Hedrich, 2018; Navarro quiroz et al., 2019; Wardowska, 2020). DNA hypomethylation in CD4^+^ T cells and B cells is a major factor contributing to SLE pathogenesis and the degree of hypomethylation is correlated with the disease activity (Javierre et al., 2010; Garaud et al., 2011; Long et al., 2016a; Wu et al., 2016a; Karagianni and Tzioufas, 2019). There are a lot of demethylated target genes identified in SLE, such as ITGAL, TET3, PRF1, MMP14, CSF3R, IFNGR2, *etc* (Javierre and Richardson, 2011; Yang et al., 2013; Zhang et al., 2013; Liu et al., 2014; Zhao et al., 2014; Wu et al., 2016b; Liao et al., 2017; Zhao et al., 2017). Various non-coding RNAs also play an important role in SLE pathogenesis through their functions in humoral and cellular immune responses (Yan et al., 2014; Honarpisheh et al., 2018). In addition, many other reports demonstrated changes in methylation or acetylation of Histone3(H3) and Histone4(H4) in SLE patients. There are significant increases of H3K4me3 in peripheral blood mononuclear cells (PBMCs) of SLE identified by chromatin immunoprecipitation linked to the microarray (ChIP-chip) approach and increased H3K4me3 is associated with the pathogenesis of the SLE (Dai et al., 2010). Increased H4 acetylation (ac) in monocytes of SLE is related to tumor-necrosis factor (TNF)αand type I interferon pathway, leading to the overproduction of proinflammatory cytokines (Sullivan et al., 2007; Zhang et al., 2010).

As a histone methylation transferase, enhancer of zeste homologue 2 (EZH2) inhibits the expression of target genes by catalyzing the formation of H3K27me3 during the development and differentiation of immune cells (Kuzmichev et al., 2002). Recent studies have identified that the expression of EZH2 is altered in SLE patients, and targeted therapy against EZH2 ameliorates the clinical performance of lupus mice (Rohraff et al., 2019; Schrezenmeier et al., 2019). Therefore, this review mainly reveals how EZH2 participates in the pathogenesis of SLE through immune equilibrium dysregulation, hoping to provide novel ideas for the treatment of SLE.

## 2 Biological functions of EZH2

**FIGURE 1:**
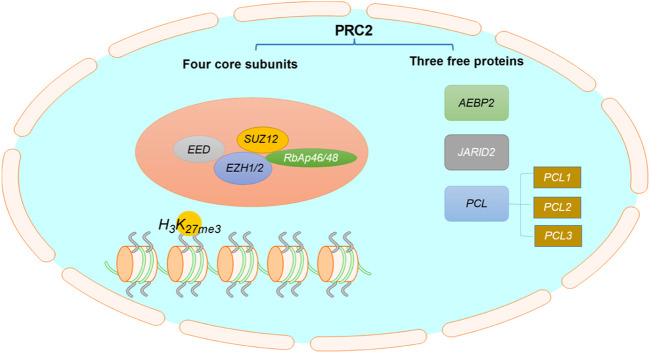
The structure of PRC2. PRC2 is composed of four core subunits: EZH1/2, EED, SUZ12, RbAp46/48, and three free proteins: AEBP2, PCLs and JARID2.

EZH2, a histone methylation transferase containing the SET domain, is a catalytic subunit of polycomb repressive complex 2 (PRC2) (Czermin et al., 2002; Kuzmichev et al., 2002). The C-terminal SET domain of EZH2 can exert the biological activity of methyltransferase (Vire et al., 2006; Poepsel et al., 2018). Structurally, PRC2 is composed of four core components (Figure 1): EZH2 or its close homolog EZH1, embryonic ectoderm development (EED), suppressor of zeste 12 (SUZ12), and retinoblastoma associated protein 46/48 (RbAp46/48, also known as RBBP7/4), as well as three free proteins: AEBP2, PCLs and JARID2 (Margueron and Reinberg, 2011; Cyrus et al., 2019; Hsu et al., 2019). EZH2 alone cannot exert its biological activity of methyltransferase, but it can play an important catalytic function when it interacts with other non-catalytic subunits such as SUZ12 and EED (Cyrus et al., 2019; Hsu et al., 2019; Zhu et al., 2019).

EZH2 exists in both the nucleus and the cytoplasm. Its most critical function is to suppress gene expression by promoting histone methylation and DNA methylation in the nucleus. EZH2 is also able to catalyze non-histones and plays a role in cytoplasmic methylation. In most cases, EZH2 exerts its biological functions through the formation of the PRC2 complex. ​In addition, EZH2 exerts some non-classical functions independent of the PRC2 complex under certain conditions.

### 2.1 Histone methylation of EZH2

The canonical biological function of EZH2 is to catalyze the lysine trimethylation of histone 3 at position 27(H3K27me3) (Vire et al., 2006). H3K27me3 is an inhibitory histone marker. The formation of H3K27me3 makes the chromosome structure denser, thereby inhibiting the expression of target genes (Czermin et al., 2002). As a histone methyltransferase, EZH2 is related to DNA replication, embryonic development, biosynthesis, cell cycle, invasiveness, and lymphocyte activation (Neo et al., 2014; Yan et al., 2016).

EZH2 is overactive in various cancer cells through functionally acquired mutations and overexpression. Previous studies have confirmed that multiple EZH2 targets in cancers (Table 1) belong to the cell cycle protein dependent kinase inhibitor (CKI) family, which negatively regulates the cell cycle (Kim and Roberts, 2016; Sha et al., 2016; Zhao et al., 2016; Tabbal et al., 2019; Wang et al., 2019). Clinical trials for EZH2 inhibitors are already underway in the field of cancer. At present, the clinical trials targeting EZH2 in tumors have indeed made great progress. There is a total of fifty-seven clinical trials regarding targeting EZH2 in cancers available on ClinicalTrials (https://clinicaltrials.gov/), nine of which have been completed (Table 2). Most new drugs targeting EZH2 are still in the preclinical phase or phase 1/2 of clinical trials, but tazemetostat was approved for the treatment of epithelioid sarcoma (ES) and preliminarily showed promising effects on follicular lymphoma (FL) (Straining and Eighmy, 2022). This is a milestone of the EZH2-targeted therapy.

**TABLE 1:** EZH2 affected target genes in cancers.

Overexpression of EZH2	Associated cancer	Target gene
	Prostate cancer	HNF1B, DAB2IP, P16, CDK4, MSMB, ADRB2, E-Cadherin, SLIT2, TIMP-2/3, EMT regulator
	Adrenocortical cancer	RRM2, PTTG1, ASE1/PRC1
	Neuroblastoma	IGFBP3
	Colon cancer	P21
	Breast cancer	CK5/6, P-cadherin, RAD51 paralogs, RUNX3, CDKN1C, FOXC1, CIITA, KLF2
	Endometrial tumor	P16, E-cadherin, sFRP1, DKK3, B-Catenin
	Melanoma	P21/CDKN1A, DCK, AMD1, WDR19
	Glioblastoma	BMPR1B
	Lung cancer	Dkk-1
	Natural killer/T-cell lymphoma	Cyclin D1
	Ovarian cancer	VASH1

**TABLE 2:** Completed EZH2-targeted clinical trials in cancers.

NCT Number	Tumor types	Interventions	Phase
NCT03456726	•Relapsed or Refractory B-cell Non-Hodgkin's Lymphoma	•Drug: Tazemetostat	Phase 2
NCT01897571	•B-cell Lymphomas	•Drug: Tazemetostat	Phase 1
	•Advanced Solid Tumors		
	•Diffuse Large B-cell Lymphoma		
	•Follicular Lymphoma		Phase 2
	•Transformed Follicular Lymphoma		
	•Primary Mediastinal Large B-Cell Lymphoma		
NCT02860286	•Mesothelioma	•Drug: Tazemetostat	Phase 2
	•BAP1 Loss of Function		
NCT02395601	•B-Cell Lymphoma	•Drug: CPI-1205	Phase 1
NCT02601937	•Rhabdoid Tumor	•Drug: Tazemetostat	Phase 1
	•INI1-negative Tumors		
	•Synovial Sarcoma		
	•Malignant Rhabdoid Tumor of Ovary		
NCT03010982	•Diffuse Large B Cell Lymphoma	•Drug: Tazemetostat and	Phase 1
	•Primary Mediastinal Lymphoma		
	•Mantle-Cell Lymphoma		
	•Follicular Lymphoma	[14C] Tazemetostat	
	•Marginal Zone Lymphoma		
	•Advanced Solid Tumors		
NCT03525795	•Advanced Solid Tumors	•Drug: CPI-1205	Phase 1
		•Drug: ipilimumab	
NCT02770391	•Prostate Cancer	•Drug: Leuprolide acetate	Phase 2
		•Drug: Apalutamide	
		•Procedure: Radical prostatectomy	
NCT03028103	•Diffuse Large B Cell Lymphoma	•Drug: Tazemetostat	Phase 1
	•Primary Mediastinal Lymphoma	•Drug: Fluconazole	
	•Mantle Cell Lymphoma	•Drug: Omeprazole	
	•Advanced Solid Tumor	•Drug: Repaglinide	
	•Marginal Zone Lymphoma		

### 2.2 DNA methylation of EZH2

DNA methylation is one of the most important epigenetic modifications, which can cause changes in chromatin structure, DNA conformation, DNA stability, and how DNA interacts with proteins, thereby regulating gene expression. EZH2 can directly control DNA methylation *via* recruiting DNA methyltransferases (DNMTs) (Vire et al., 2006), facilitating the binding of DNMTs to EZH2 target genes. EZH2 is also required for DNA methylation of EZH2 target promoters, supported by EZH2 controlled CpG methylation through direct physical contact with DNMTs. EZH2 serves as a recruitment platform for DNMTs, indicating that histone modification and DNA methylation jointly regulate the epigenetic state (Cedar and Bergman, 2009).

### 2.3 Non-histone methylation of EZH2

In addition to the canonical role of EZH2 as a histone methyltransferase in nucleus, it is clear that EZH2 can also methylate non-histones in the cytoplasm and cell nucleus. The non-histone methylation function of EZH2 also depends on the formation of PRC2 complex. EZH2 can regulate cell migration, invasion and adhesion by mediating the methylation of cytoplasmic proteins. Cytoplasmic EZH2 can regulate actin polymerization and cell mobility in various cell types by methylating Vav1 protein (Su et al., 2005). EZH2 can regulate the migration and adhesion dynamically in neutrophils and dendritic cells (DCs) by direct methylation of cytosolic talin (Loh et al., 2018). EZH2 disrupts the link between actin and talin by polymerizing the actin cytoskeleton (F-actin) and blocking the binding motif of talin, thereby aggravating the migration and invasion of cells. Moreover, cytoplasmic PRC2 can promote the proliferation of CD4^+^T cells and CD8^+^T cells by methylating Vav1 and Vav1 associated protein Nck (Dobenecker et al., 2018). In the nucleus, EZH2 can also methylate transcription factors, such as GATA4 and RORα, leading to functional repression (He et al., 2012) and proteasomal degradation (Lee et al., 2012). Furthermore, EZH2 can interact with STAT3 and directly methylate STAT3, resulting in enhanced nuclear localization and chromatin of STAT3, thereby exacerbating breast cancer (Yang et al., 2021; Zhao et al., 2021).

### 2.4 Non-canonical functions of EZH2

Apart from being designated as a methyltransferase, EZH2 can also play an important role independent of PRC2 in certain situations. EZH2 directly methylates the transcription factor PLZF of the natural killer T (NKT) cell lineage, leading to its ubiquitination and subsequent degradation, which impacts the development of the immune system (Vasanthakumar et al., 2017). EZH2 can also be converted into a transcriptional activator, as reported in a natural killer T cell lymphoma (NKTL) disease model (Yan et al., 2013; Yan et al., 2016). EZH2 can directly promote the transcription of cyclin D1 independent of its methyltransferase activity, inducing the growth of NK tumor cells in NKTL (Yan et al., 2013). The phosphorylation of EZH2 by janus kinase 3 (JAK3) promotes the PRC2 complex’s dissociation, switching EZH2 to a transcriptional activator, associated with a set of upregulated genes involved with DNA replication, cell cycle, biosynthesis, stemness and invasiveness (Yan et al., 2016). JAK3 inhibitors can significantly reduce the growth of NKTL cells in an EZH2 phosphorylation-dependent manner, while drugs that inhibit EZH2 methyltransferase activity have no such effect, which provides a new option for the treatment. EZH2 enhances breast cancer initiation by directly binding to the NOTCH1 promoter and activates NOTCH1 signaling independent of its catalytic H3K27me3 activity (Gonzalez et al., 2014). EZH2 exerts distinct non-histone methyltransferase roles in ER-negative and positive breast cancers. In ER-negative breast cancer, EZH2 physically interacts with RelA/RelB to promote the expression of NF-κB targets. Interestingly, EZH2 acts inversely in ER-positive luminal breast cancer cells and suppresses the expression of NF-κB target genes by interacting with ER and directing repressive histone methylation to its promoters (Lee et al., 2011). Consequently, EZH2 functions in breast cancer as a double-sided molecule that, acts as a transcriptional activator or repressor of NF-κB targets depending on the cellular context. What’s more, EZH2 interacts directly with estrogen receptor and β-catenin through transactivation in its two N-terminal domains, thereby linking estrogen and Wnt signaling pathways to promote cell cycle progression and ER-positive breast cancer progression (Shi et al., 2007). In addition, EZH2 acts as a transcriptional activator to activate androgen receptor (AR) gene transcription by directly occupying its promoter to promote prostate carcinogenesis (Kim et al., 2018). Another non-canonical function of EZH2 is to phosphorylate H2BY37 and induce transcriptional activation of ATG in colon cancer cells (Liu et al., 2020).

**FIGURE 2:**
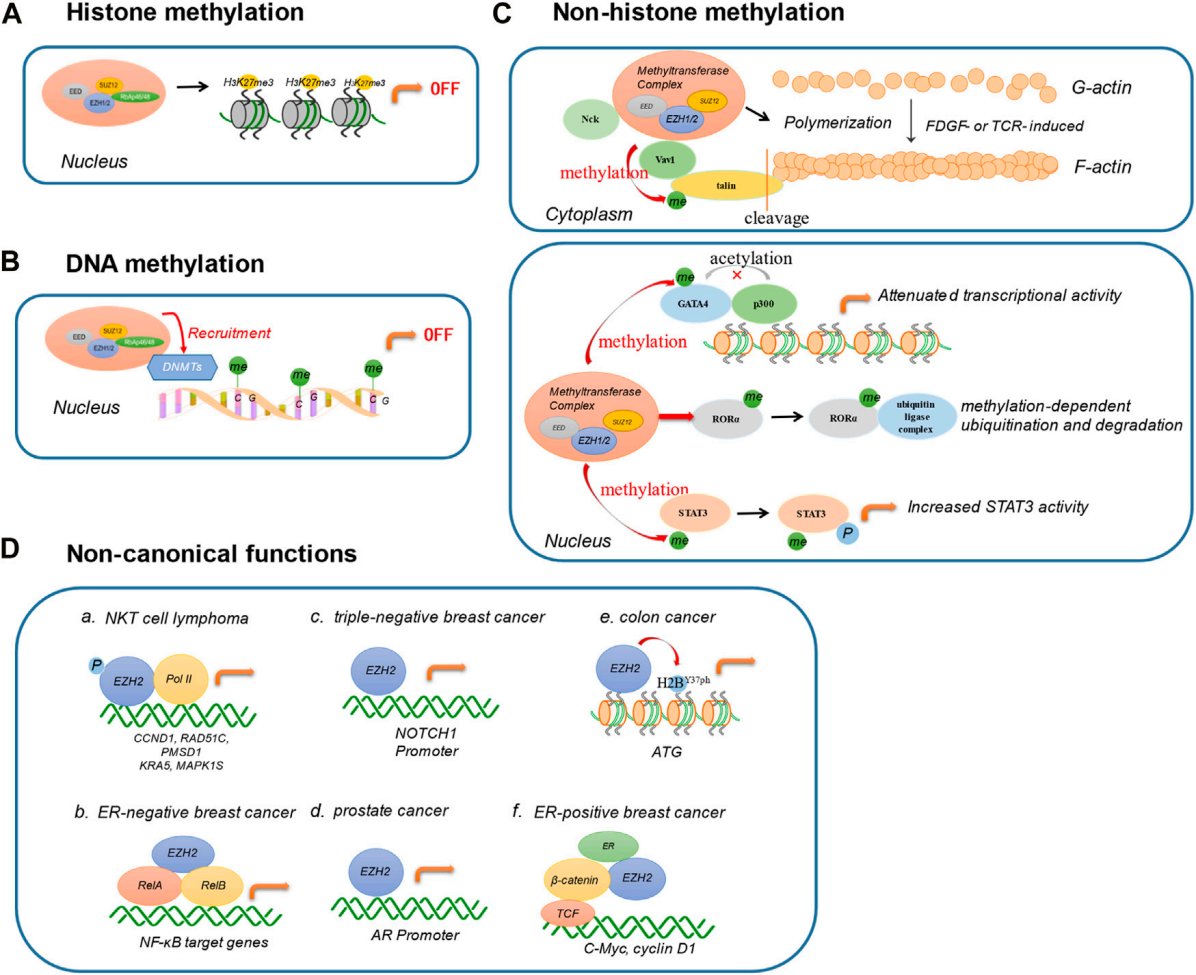
Biological functions of EZH2. **(A)** Histone methylation. ​EZH2 catalyzes the lysine trimethylation of histone 3 at position 27. **(B)** DNA methylation. EZH2 directly controls DNA methylation *via* recruiting DNA methyltransferases (DNMTs). **(C)** Non-histone methylation. EZH2 regulates cell migration, invasion, and adhesion by mediating the methylation of cytoplasmic proteins, including Vav1, Nck, and talin. In the nucleus, EZH2 can also methylate transcription factors for diverse functions, such as GATA4, RORα, and STAT3. **(D)** Non-canonical functions. EZH2 exerts non-canonical functions in a PRC2-independent complex. a. In NKT cell lymphoma, the phosphorylation of EZH2 directly binds with Pol II involved in DNA replication, cell cycle, biosynthesis, stemness, and invasiveness. b. In ER-negative breast cancer, EZH2 interacts with RelA/RelB to promote the expression of NF-κB targets. c. In triple-negative breast cancer, EZH2 activates NOTCH1 signaling by directly binding to NOTCH1 promoter. d. EZH2 combines with AR promoter in prostate cancer. e. EZH2 regulates H2B phosphorylation and then induces the transcription activation of ATG in colon cancer. f. EZH2 interacts directly with estrogen receptor and β-catenin, promoting ER-positive breast cancer progression. (DNMTs: DNA methyltransferases; ER: estrogen receptor; AR: androgen receptor).

Collectively, EZH2 has multiple biological functions and plays a different role in different cells and diseases (Figure 2). With the in-depth study of EZH2, it will help us to better understand the function of EZH2 and provide new ideas for targeting EZH2 in tumor therapy.

## 3 The role of EZH2 in the immune system

The immune system is a dynamic and fine-tuned buffering system, containing both effector and regulatory components. Effector T cells, activated B cells and M1 macrophages induce immune response and inflammation by generating proinflammatory cytokines, chemokines and antibodies. In contrast, regulatory T cells, regulatory B cells and M2 macrophages suppress the immune response and mediate immune tolerance through producing suppressive mediators (O’neill et al., 2016). ​Numerous cells and molecules function cooperatively in both innate and adaptive immunity. The homeostasis of immune system is the cornerstone of the body’s fight against infections and tumors, and an imbalance between the effector and regulatory components of the immune system can lead to autoimmune diseases. EZH2 affects the homeostasis of the immune system through multiple aspects (Huntington and Gray, 2018; Horwitz et al., 2019). For example, EZH2 mediates the development, differentiation, activation, proliferation and cytokine secretion of immune cells (Nutt et al., 2020). In the following part, we will describe the effects of EZH2 on innate immunity and adaptive immunity.

### 3.1 The effects of EZH2 on adaptive immunity

Adaptive immunity, mediated by T cells and B cells, is induced by certain antigens and is antigen specific. Compared with innate immunity, adaptive immunity has many characteristics, such as self-limitation, self-tolerance and immune memory. The development and differentiation of adaptive lymphoid cells require EZH2. The following section will cover detailed mechanisms underlying the EZH2-mediated-regulation of T and B cells in the adaptive immune response.

#### 3.1.1 T lymphocytes

T cells occupy a central position in the adaptive immune response by mediating cellular immune responses and humoral immune responses against thymus-dependent antigens. The differentiation and function of different T cell subsets are accompanied by EZH2-mediated transcriptome remodeling.

Helper T cells assist T and B cell immune responses. Stimulated by different antigens and cytokines, naive CD4^+^T cells can differentiate into different effector cells, such as Th1, Th2, Th17, Th22, and follicular helper T cells (Tfh). EZH2 plays a vital role in the differentiation and maturation of different subpopulations of CD4^+^T cells, but its role is still controversial and the mechanism is not completely clear (Koyanagi et al., 2005; Jacob et al., 2008; Wei et al., 2009; Kanno et al., 2012; Tumes et al., 2013; Tong et al., 2014; Zhang et al., 2014; Dupage et al., 2015; Yang et al., 2015; Karantanos et al., 2016; Li et al., 2018; Chen et al., 2020; Zhu et al., 2020).

Th1 cells express interferon (IFN)-γ/transcription factor T-bet and participate in the immune response towards intracellular pathogens. An increment in Th1 can cause autoimmune diseases. Th2 cells express interleukin (IL)-4 and Gata3 and limit worm infection, involved in allergies and parasitic infections. EZH2 binds directly to Tbx21 (encoding T-bet) and Gata3 (encoding Gata3), inhibiting the transcription activity of two TFs, and ultimately suppressing the differentiation of naive CD4^+^T cells into Th1 and Th2 cells (Koyanagi et al., 2005; Tumes et al., 2013; Zhang et al., 2014). Besides, EZH2 inhibits the differentiation of Th1 and Th2 cells by direct epigenetic labeling and silencing of genes that encode lineage-specific cytokines, such as Ifng and II13 (Koyanagi et al., 2005; Tumes et al., 2013; Zhang et al., 2014). Moreover, the lack of EZH2 accelerates the death of effector Th cells through death receptor-mediated external and internal apoptotic pathways (Zhang et al., 2014). LncRNA GAS5 can inhibit Th1 differentiation and promote Th2 differentiation through EZH2 and T-bet in allergic rhinitis (Zhu et al., 2020). Paradoxically, EZH2 can also induce Th1 cell differentiation by increasing T-bet stability and inducing the secreting of Th1 cytokines (Jacob et al., 2008; Tong et al., 2014).

Tfh cells produce IL-21 and help B cells participate in humoral immunity. The increase in Tfh causes antibody-related autoimmune diseases. Tfh differentiation is a multi-stage process involving many transcription factors that drive the specification and functional maturity of the Tfh lineage (Liu et al., 2013; Crotty, 2014). In early committed Tfh cells, the expression of EZH2 and H3K27me3 modification is upregulated. EZH2 specifically stabilizes the chromatin plasticity of a set of genes critical for Tfh fate commitment, notably B-cell lymphoma 6 (Bcl6), to direct Tfh cell commitment in a mouse model of acute lymphocytic choriomeningitis virus (LCMV) infection (Chen et al., 2020). Contrary to the well-known function as a chromatin repressor, EZH2 indirectly maintains the chromatin accessibility and reinforces the expression of Bcl6 (Baumjohann et al., 2011; Choi et al., 2013). EZH2 can inhibit the expression of p19Arf in the Cdkn2a locus, thereby promoting Tfh lineage stereotypes and functional maturity by shielding Tfh cells from Bcl6 inhibition, and promoting Tfh cell survival by protecting them from p53 induction (Li et al., 2018). These findings determine that EZH2 is an integrator of epigenetic and transcriptional regulatory mechanisms that can program the fate, survival and functional maturity of Tfh.

Th17 cells produce IL-17, express Rorc, and defend against extracellular bacteria and fungi. Th17 cells play an important role in autoinflammation and autoimmunity. Th22 cells produce IL-22, participating in body defense functions. Excessive Th22 causes inflammatory diseases and autoimmune diseases (Kanno et al., 2012; Jia and Wu, 2014). It is generally believed that Th17 and Th22 are closely related to the occurrence of autoimmune diseases, but there is little research about the role of H3K27me3 in Th17/22. EZH2 suppresses Th17 differentiation (Yang et al., 2015). Inhibition of the H3K27me3 demethylase KDM6A/B leads to an overall increase in the inhibitory H3K27me3 histone markers, resulting in the inhibition of the key transcription factor RORγt during Th17 differentiation, which in turn leads to metabolic reprogramming (Cribbs et al., 2020). The Th22 cell-derived IL-22 activates STAT3 and targets PRC2 to induce colon cancer cell proliferation *via* H3K27me3 (Sun et al., 2016).

Tregs negatively regulate the immune response by inhibiting the activation and proliferation of CD4^+^and CD8^+^T cells to maintain immune homeostasis. However, Tregs exhibit plastic differentiation and have specific functions of Th cells, such as the secretion of Th-related cytokines and the expression of specific transcription factors in Th cells in autoimmune diseases (Qiu et al., 2020). Foxp3 is specifically expressed in Tregs, which is essential for the differentiation and function of Tregs (Ziegler, 2006; Josefowicz et al., 2012). The interaction between Foxp3 and EZH2 only occurs in activated Treg cells (Arvey et al., 2014). Foxp3 can bind to EZH2 to inhibit transcription, thereby regulating the Treg cell phenotype (Kwon et al., 2017). In rheumatoid arthritis patients, downregulation of EZH2 in CD4^+^ T cells suppresses Foxp3 transcription by down-regulating RUNX1 and up-regulating SMAD7, finally leading to inhibition of Treg differentiation (Xiao et al., 2020). EZH2 mediates Tregs activation *via* IFN-γ and IL-4 downregulation, along with the consequent inducing of Foxp3 (Karantanos et al., 2016). Also, the lack of EZH2 can lead to a reduction in the number of Tregs and impaired function (Yang et al., 2015). Follicular regulatory T (Tfr) cells are a subset of regulatory T cells that control the production of antibodies by inhibiting the help of B cells mediated by Tfh. Foxp3 and EZH2 are required for the maintenance of Tfr cellular transcriptional programs, and the absence of Foxp3 or EZH2 may lead to a weakened inhibitory effect of Tfr cells on B cell responses (Hou et al., 2019).

The CD8^+^ subset of T cells proliferates and differentiates into cytotoxic T lymphocytes (CTLs), which express cytotoxic granules and kill infected cells. EZH2 can regulate the proliferation and apoptosis of naive CD8^+^T cells (Karantanos et al., 2016). MiR-26a is a negative regulator of CTL function in the tumor microenvironment (TME). EZH2 is negatively correlated to miR-26a in CTL, and inhibition of EZH2 in CTL impairs the function of secreting granzyme B/IFN-γin TME (Long et al., 2016b). The absence of EZH2 results in increased expression of cyclin-dependent kinase inhibitors Cdkn2a (p16 and Arf) and Cdkn1c (p57) in activated naive CD8^+^T cells (Chen et al., 2018), thereby inhibiting cell proliferation.

#### 3.1.2 B lymphocytes

The main functions of B cells are to produce antibody mediating humoral immune response, present antigens and produce cytokines to participate in immune regulation. Multiple stages of B cell development in bone marrow and B cell immune response outside the bone marrow have been affected by EZH2. In bone marrow, EZH2 is necessary for the normal maturation from pro-B to pre-B stage through different mechanisms. In the early development of B cells, the lack of EZH2 causes B cell development to stay in the stage of differentiation from pro-B cells to pre-B cells (Su et al., 2003). The major mechanism is that EZH2 can regulate the heavy chain VDJ rearrangement and promote the formation of pre-BCR, so that pro-B cells can develop to the pre-B cell stage. At the same time, the effect of EZH2 on the expression of Igκ chain also directly affects the early differentiation of B cells in bone marrow (Mandal et al., 2011). However, EZH2 does not affect the survival of mature B cells in the spleen, and the ratio of marginal zone B cells to follicular B cells, which indicates that EZH2 does not affect the maintenance of B cell homeostasis in surrounding lymphoid tissues (Guo et al., 2018). Outside the bone marrow, EZH2 mediates both T-cell-dependent and T-cell-independent immune responses. The expression of EZH2 in naïve B cells in the resting state is extremely low, and the expression is rapidly increased in activated B cells and germinal center (GC) B cells (Su et al., 2003; Guo et al., 2018). EZH2 promotes the generation of GC while maintaining GC function through the following mechanisms (Velichutina et al., 2010; Beguelin et al., 2013; Caganova et al., 2013; Beguelin et al., 2016; Beguelin et al., 2017). Firstly, EZH2 can promote the rapid division and proliferation of GC B cells by inhibiting CKI to promote the formation of GC. Secondly, EZH2 can cooperate with the anti-apoptotic protein BCL2 to inhibit cell apoptosis, and then maintain the GC morphology. Lastly, in the GC B cells, EZH2 can inhibit the expression of terminal differentiation-related genes, sustain the germinal center B cell phenotype, and then maintain the germinal center reaction. B cell–specific deletion of EZH2 leads to a loss of GC formation, thereby leading to defects in the formation of plasma cells (PCs). Moreover, EZH2 promotes the final differentiation of peripheral mature B cells into plasma cells *via* extra-follicular immune response or T cell-independent immune response (Guo et al., 2018). The expression of EZH2 in PCs is further increased than in activated B cells. EZH2 controls transcriptional changes during plasma cells differentiation controlling several B and plasma cell genes through PRC2 and H3K27me3 dependent mechanisms (Scharer et al., 2018; Herviou et al., 2019).

EZH2 affects not only the quantity but also the quality of PCs. EZH2-defficient PCs display an impaired function of antibody secretion. The main mechanism is that EZH2 affects the unfolded protein response in PCs and affects the normal metabolic functions of PCs, including oxidative phosphorylation and glycolysis (Guo et al., 2018).

### 3.2 The effects of EZH2 on innate immunity

Innate immunity is the initial response to microbes that prevents, controls, or eliminates the host’s infection. Innate lymphoid cells (ILCs), include natural killer (NK) cells, DCs, mast cells, and ILC groups 1–3. Relatively, little research has been done on the role of EZH2 in NK cells, DCs.

#### 3.2.1 NK cells

NK cells can directly kill infected cells by releasing particles containing perforin and granzyme, or indirectly activate phagocytic microbes to phagocytic macrophages through IFN-γ release in the early stages of infection, thereby inhibiting virus replication (Vivier et al., 2008; Vivier et al., 2011). EZH2 regulates NK cells development and functions through its histone methyltransferase activity. In hematopoietic stem and progenitor cells (HSPC), EZH2-inhibitors gave rise to increased NK precursors, leading to up-regulation of IL-15R (CD122) and NKG2D activated receptors, which were associated with enhanced NK cell expansion and cytotoxicity against tumor cells (Yin et al., 2015a). And EZH2-inhibitors treatment enhances NK cell eradication of hepatocellular carcinoma cells in an NKG2D ligand-dependent manner (Bugide et al., 2018). These findings may provide insights into the contribution of epigenetic regulation to the origin of NK cells, and reveal that EZH2 inhibitors can directly or indirectly inhibit tumor growth by mobilizing NK cells.

#### 3.2.2 Dendritic cells

DCs are the most powerful antigen-presenting cells (APCs) in the body at the center of initiating, regulating, and maintaining the immune response, which can efficiently ingest, process and present antigens (Macri et al., 2018). The abnormal regulation of their homeostasis and function is involved in the occurrence of autoimmune diseases (Kaewraemruaen et al., 2019). EZH2 is necessary to activate DCs, mediating epigenetic modification in allergen immunotherapy (Li et al., 2019). EZH2 controls cell adhesion and migration in DCs through methylation of the cytoplasmic regulatory protein talin (Gunawan et al., 2015). And EZH2 controls skin tolerance by regulating different subsets of skin DCs through methylation of talin1 (Loh et al., 2018).

## 4 EZH2 plays a role in systemic lupus erythematosus

Inheritance, epigenetics, sex hormones, infection, environment and other factors are all involved in lupus pathogenesis. Immune factors and non-immune factors are all closely related to the occurrence of lupus. Cell populations of both innate immune system (monocytes, macrophages and dendritic cells, natural killer cells, and innate lymphoid cells) and adaptive immune system (T cells and B cells) display hyper-responsiveness or hypo-responsiveness through epigenetic reprogramming in SLE. Abnormal proliferation and activation of B cells in SLE patients lead to peripheral immune tolerance deficiency and terminal differentiation of autoreactive plasma cells, which in turn produce autoantibodies (Barnas et al., 2019). T cells promote autoimmune responses and amplify systemic inflammation in SLE by producing inflammatory cytokines and assisting B cells to secret antibodies (Chen and Tsokos, 2021). EZH2 impacts the delicate balance of immune homeostasis in SLE patients by regulating T, B, and other immune cells (Rohraff et al., 2019). Compared with controls, SLE patients present an increased expression of EZH2 in PBMCs (Wu et al., 2021), neutrophils, monocytes, B cells, and CD4^+^T cells (Rohraff et al., 2019), which may involve the activation of type I interferon signaling pathway and Notch signaling pathway (Amsen et al., 2004; Ciofani and Zuniga-Pflucker, 2005; Cruickshank and Ulgiati, 2010; Zhao et al., 2016; Huang et al., 2017; Arima et al., 2018; Wu et al., 2021). MRL/lpr mice treated with EZH2 inhibitors exhibit lowered anti-dsDNA antibodies, decreased inflammatory cytokines, improved symptoms, and reduced mortality, indicating that EZH2 is a promising therapeutic target for lupus.

### 4.1 EZH2 is involved in T lymphocyte dysfunction of systemic lupus erythematosus

The dysfunction of CD4^+^T cells is critical in the pathogenesis of lupus (Rapoport and Bloch, 2012; Yin et al., 2015b; Dolff et al., 2019). Cytokine production, complement activation, and clonal B cell activation secondary to CD4^+^ T cellular immune disorders in lupus lead to the overproduction of autoantibodies and tissue damage (Comte et al., 2015).

EZH2 and H3K27me3 levels are elevated in CD4^+^ T cells of SLE. Over-activated rapamycin complex 1 (mTORC1) signaling and elevated glycolysis down-regulate miR-26a and miR-101 in patients with SLE, resulting in increased expression of their target EZH2 (Barnas et al., 2019; Chen and Tsokos, 2021). EZH2 upregulation in CD4^+^ T increases DNA methylation of the F11R gene encoding junctional adhesion molecule A (JAM-A), resulting in an imbalance of T cells and increased adhesion to endothelial cells in SLE patients. (Tsou et al., 2018). Inhibition of EZH2 and mTOR or glycolysis may have a particular therapeutic effect on SLE.

Increased BCL-6 on the miR-142 promoter up-regulates H3K27me3 (repressive mark) and down-regulates H3K9/14ac (activating mark), resulting in a decrease in miR-142-3p/5p expression, which leads to CD4^+^T cell over-activity in SLE (Ding et al., 2012; Ding et al., 2019). Another study (Sawalha, 2019) suggests that BCL-6 and EZH2 cooperate to inhibit miR-142-3p/5p expression in SLE CD4^+^T cells through epigenetic modification. MiR-142-3p/5p is anti-inflammatory, and when suppressed, CD4^+^T cells overexpress inflammatory molecules, including CD40L, ICOS, and IL-21, leading to an immune imbalance.

CD8^+^T cells exhibit decreased effector function and cytolytic activity in SLE, leading to an increased infection rate (Kis-toth et al., 2016; Comte et al., 2017). However, the involved mechanisms are not understood. CD38 is elevated in CD8^+^ T cells in SLE patients with increased rates of infections. The degranulation, secretion of granzyme and perforin of CD8^+^CD38^high^T cells are all decreased. CD38 leads to increased acetylation of EZH2 by inhibiting the acetylase Sirtuin1. Acetylated EZH2 leads to a decrease in the expression of RUNX3, a transcription factor associated with CD8^+^ T cells, which impairs the function of CD8^+^ T cells (Katsuyama et al., 2020). And inhibiting EZH2 can restore the cytolytic function of CD8^+^CD38^high^ T cells, which may provide promise to overcome the incidence of infections in SLE patients (Chakraborty and Mehrotra, 2020). But inhibiting EZH2 can also impair the suppressive functions of Tregs, which can lead to spontaneous autoimmunity (Dupage et al., 2015). Although the inhibition of EZH2damages the function of Tregs, animal experiments confirm that the application of EZH2 inhibitors in lupus mice can inhibit autoimmunity and improve lupus-related symptoms. Therefore, from a global perspective, it can be considered that EZH2 mediates lupus autoimmunity and EZH2 inhibitors can improve the destruction of immune tolerance. However, there is no research exploring the changes in EZH2 expression in Treg and the impact of EZH2 on the Treg function in SLE. This will be the direction of future research on the pathogenesis of lupus.

### 4.2 EZH2 is involved in systemic lupus erythematosus with B lymphocyte dysfunction

Abnormal B cells also play a vital role in the pathogenesis of SLE. Dysregulation of B cell transcription factors, cytokines and B cell-T cell interaction can result in aberrant B cell maturation and autoantibody production (Yap and Chan, 2019). The peripheral immune deficiency of B cells may be related to the activation of toll-like receptors (TLR) pathway in SLE (Tsokos et al., 2016). EZH2 is increased in B cells and positively correlates with disease activity and autoantibody production. EZH2 is almost exclusively increased in plasmablasts, but not in naive and memory B cells in lupus (Schrezenmeier et al., 2019). mTORC1 is activated in B cells and regulates plasma cell differentiation in SLE patients. In the presence of methionine, Syk, and mTORC1 activation can co-induce the expression of EZH2 (Zhang et al., 2020). EZH2 induces H3K27me3 at the BTB and CNC homology 2 (BACH2) locus and inhibits BACH2 expression, thereby causing the expression of B lymphocyte induced maturation protein 1(BLIMP1) and X-box binding protein 1 (XBP1) and differentiation of plasmablasts. These results indicate that methionine activates signaling by controlling B cell immune metabolism and plays an important role in the differentiation of B cells into plasmablasts through epigenomic modification. And EZH2-inhibitor suppresses autoantibody production and GC formation in bm12 induced lupus-like chronic graft-versus-host disease (cGVHD) (Zheng et al., 2020). Therefore, we speculate that EZH2 may promote B cell proliferation and inhibit B cell apoptosis by regulating cyclin-dependent kinase inhibitors in SLE, thereby abnormally proliferating plasma cells, leading to the production of autoantibodies.

The pathogenesis of lupus also involves a variety of ILCs. The number of NK cells in SLE patients is reduced and their function is impaired. (Hervier et al., 2011). EZH2 inhibits the development of NK cells, as described in the third part. However, there is no research investigating whether NK cell reduction in SLE patients is associated with elevated EZH2 levels. This may be worth further research to provide new ideas for uncovering the pathogenesis of lupus. And more notably, EZH2 may participate in the pathogenesis of SLE by regulating differentiation and functions of ILCs.

**FIGURE 3:**
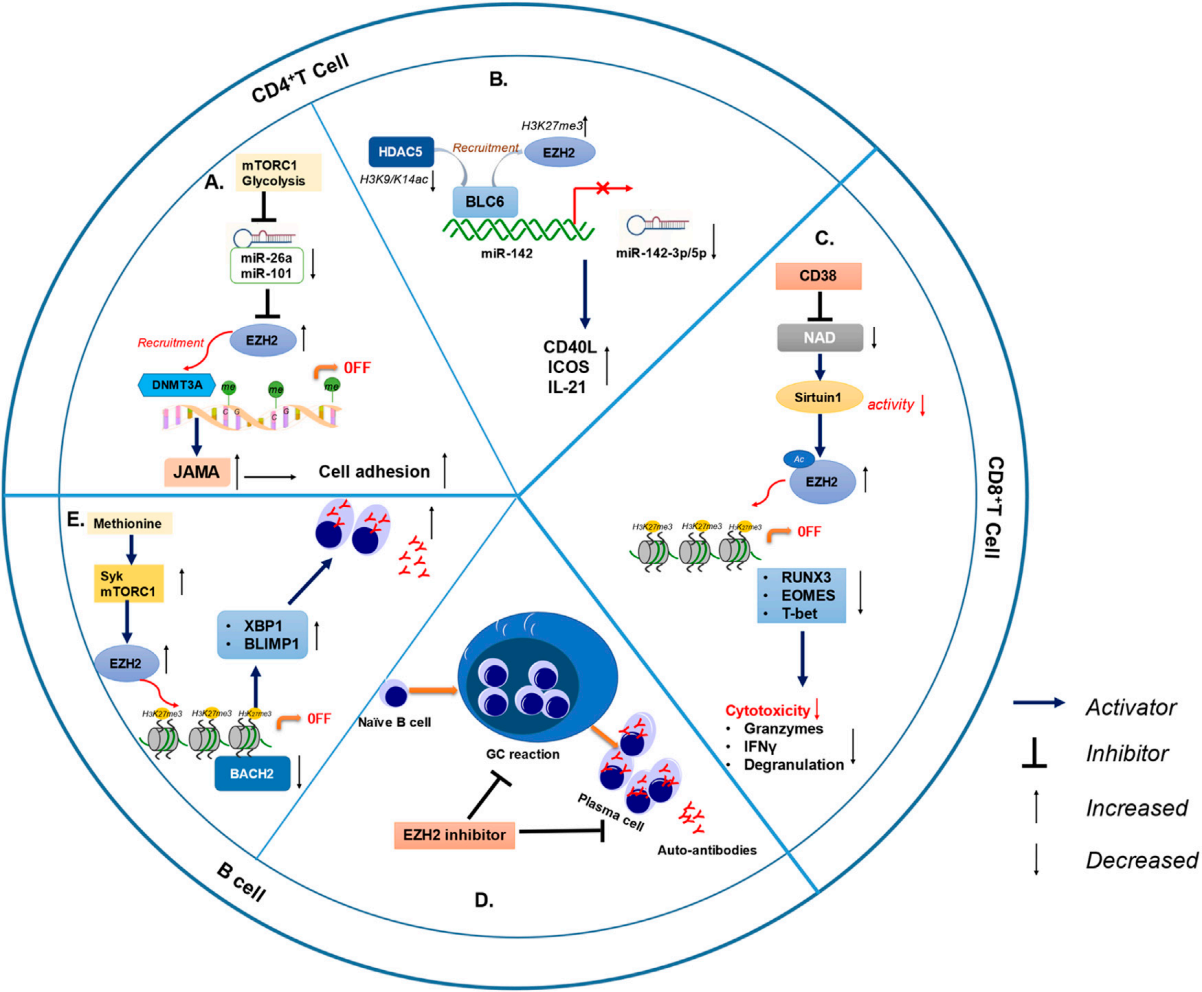
EZH2 is involved in lupus pathogenesis through different immune cells. **(A)** MiR-26 and miR-101 was inhibited by higher glycolysis and mTOR1, limiting post-transcriptional suppression of EZH2 and leading to increased EZH2 levels in SLE CD4^+^ T cells. Elevated EZH2 result in increased JAM-A expression and CD4^+^ T cell adhesion of lupus. **(B)** BCL-6 recruited EZH2 and HDAC5, resulting in increased H3K27me3 (repressive mark) and decreased H3K9/K14ac (activating marks), which lead to epigenetic repression of miR-142 and reduced miR-142-3p/5p expression in lupus CD4^+^T cells. In addition, CD4+T cells secret more inflammatory molecules when miR-142-3p/5p was decreased, including CD40L, ICOS and IL-21. **(C)** The CD38/NAD/Sirtuin1/EZH2 axis increased the methylation of RUNX3, EOMES, and T-bet, in turn, reducing cytolytic CD8^+^ T cell functions in SLE patients. **(D)** EZH2 inhibitor reduced autoantibody production and GC formation in bm12 induced lupus-like cGVHD. **(E)** Syk and mTORC1 activation synergistically induced EZH2 expression in the presence of methionine. EZH2 induced H3K27me3 at the BACH2 locus and repressed BACH2 expression, thereby inducing increased BLIMP1 and XBP1 expression and plasmablast differentiation. (mTOR1: mechanistic target of rapamycin complex 1; GC: germinal center; cGVHD: chronic graft *versus* host disease).

In general, EZH2 mediates the development of SLE through the disruption of immune homeostasis in a variety of ways (Figure 3; Table 3). The role of EZH2 in CD4^+^ T cells and B cells has been widely reported, and it is likely that EZH2 in these cells has a greater impact on the pathogenesis of SLE than other immune cells. EZH2 expression is elevated in CD4^+^ T cells in lupus, leading to increased cytokine secretion and increased T cell adhesion. EZH2 is associated with decreased CD8 cytotoxic function in lupus-infected patients. More importantly, EZH2 can inhibit the expression of BACH2 and promote the differentiation of plasma cells, which is closely related to autoantibody production. The application of EZH2 inhibitors improved lupus-like symptoms in MRL/lpr mice, lowered the production of type I interferon in NZW/NZB F1 mice and reduced GC formation and autoantibody secretion in bm12 induced lupus-like cGVHD. These studies verified that EZH2 is essential in the pathogenesis of SLE by governing the function of multiple immune cells. Although multiple medications have been approved for the treatment of SLE, there is still a proportion of patients with poor prognosis. Therefore, the development of new drugs for lupus is of great significance. Clinical studies on EZH2 inhibitors in the field of oncology have confirmed the efficacy and safety of EZH2 inhibition therapy. In addition, a variety of medications treating SLE such as rituximab, cyclophosphamide, and methotrexate were originally used in hematological malignancy and other cancers field. Based on the research of EZH2 in lupus patients and mouse models, EZH2 inhibition may become a promising treatment for SLE.

**TABLE 3:** The role of EZH2 in immune dysregulation of SLE pathogenesis.

Research objects		Immune cells	Expression	Related pathogenesis	References
SLE patients		CD4^+^T cells	Decreased		Hu et al. (2008)
		CD4^+^T cells		EZH2 was positively correlated methylation sites relevant to disease activity and flares	Coit et al. (2016)
		CD4^+^T cells	Increased	Increased EZH2 in CD4^+^T cells lead to elevated JAM-A and CD4^+^ T cell adhesion	Tsou et al. (2018)
		CD4^+^T cells		BCL-6 and EZH2 cooperated to inhibit the express of miR-142-3p/5p, causing increased cytokine production	Ding et al. (2019)
		Monocytes, neutrophils, B cells and CD4^+^ T cells	Increased		Rohraff et al. (2019)
		PBMCs	Increased	​EZH2 overexpression was associated with the activation of the IFN-I signaling pathway	Wu et al. (2021)
		CD4^+^ T cells	Increased	Activation of mTORC1 and increased glycolysis induced an elevated EZH2 level	Zheng et al. (2020)
		CD8^+^T cells		CD8CD38^high^T cells exhibited reduced cytotoxicity through the NAD^+^/Sirtuin1/EZH2 pathway	Katsuyama et al. (2020)
		B cells	Increased	EZH2 was increased in plasmablasts but not in memory and naïve B cells	Schrezenmeier et al. (2019)
		B cells	Increased	Elevated EZH2 induced plasmablast differentiation *via* BACH2 repression, closely related to metabolism	Zhang et al. (2020)
Lupus mice	MRL/lpr mice			EZH2 inhibitor improved survival, reduced anti-dsDNA antibody production, lowered renal involvement, lymphoproliferation and decreased cytokines production	Rohraff et al. (2019)
	NZW/NZB F1 mice			EZH2 inhibitor alleviated lupus nephritis	Wu et al. (2021)
	The mouse model of lupus-like cGVHD	B cells, CD4^+^ T cells	Increased	EZH2 inhibitor suppressed autoantibody production and GC formation	Zhen et al. (2020)

## 5 Conclusion

As an epigenetic regulator, EZH2 has various biological functions, the most important of which is to play the role of histone methyltransferase. EZH2 promotes the initiation and maintenance of immune and inflammatory responses by involving in the development, activation and differentiation of various immune cells. EZH2 is abnormally elevated in lupus and is associated with immune homeostasis imbalance and disruption of autoimmune tolerance in SLE patients. Therefore, EZH2 may serve as an important biological marker for the diagnosis and target for the treatment of SLE.
